# Rebuilding the health sector in Gaza: alternative humanitarian voices

**DOI:** 10.1186/s13031-024-00599-0

**Published:** 2024-05-31

**Authors:** Karl Blanchet, Martine Najem, Lina Shadid, Rouba Ali Fehmi, Fawzi Al Hammouri, Ghassan Saed, Khaled J. Saleh, Mosab Nasser, Nidal Moukaddam, Jonathan Polonsky, Omar Lattouf

**Affiliations:** 1 Geneva Centre of Humanitarian Studies, Faculty of Medicine, University of Geneva, Geneva, Switzerland; 2 American University of Beirut, Beirut, Lebanon; 3 PWC Lead Healthcare, Dubai, United Arab Emirates; 4 University of Michigan, Ann Arbor, Michigan USA; 5 NAAMA, Amman, Jordan; 6 FAJR Scientific, Ann Arbor, USA; 7 Psychiatry Baylor College of Medicine, Waco, USA; 8 Emory University School of Medicine, NAAMA, Atlanta, USA

## Abstract

In November 2023, a variety of disparate health organizations formed an international coalition to consolidate efforts and develop collaborative strategies in response to the increasing critical healthcare challenges caused by the recent war in Gaza. The coalition includes medical and public health experts, humanitarian practitioners, academics, and health policy-makers from across the world. Their membership has not much to do with mainstream humanitarian organisations. It is lead by the diaspora from the region. Their vision is the long-term reconstruction of the health system in Gaza while responding the most urgent needs. This collective effort will require explicit efforts to speak with one voice and avoid duplication. This collective movement may be an orginal initiative that may be able to beat the expected international donor fatigue.

In November 2023, a variety of disparate health organizations formed an international coalition to consolidate efforts and develop collaborative strategies in response to the increasing critical healthcare challenges caused by the recent war in Gaza. The coalition includes medical and public health experts, humanitarian practitioners, academics, and health policy-makers from across Palestine, the Middle East, Australia, Japan, Indonesia, South Africa, New Zealand, Canada, USA and Europe, some of whom have been working in Gaza for over twenty years. This collective of organisations and individuals has distinctive characteristics: they are mostly non-profit organisations and all work in, or are connected to, Palestine. The membership base comprises health professionals who volunteer their time, for example conducting medical missions inside the Gaza Strip, or organising medical evacuations for Palestinian patients in need of specialised care, amongst many other activities [1]. The movement was initiated by the National Arab American Medical Association (NAAMA), the oldest Arab American medical association in the US, active for fifty years in medical, educational and humanitarian support services to Palestine and other countries in the Middle East region [2].

On February 7th 2024, the coalition convened the “1st International Conference to Rebuild the Health Sector in Gaza” in Amman, Jordan to join forces and plan collective humanitarian assistance with a long-term view to “restore, rebuild and strengthen the health sector in Gaza” [3]. NAAMA’s membership and outreach to thousands of healthcare providers worldwide brought together over five hundred onsite participants for this conference, with two hundred more joining online. Over 150 organizations were represented, including the Jordan Medical Association, Jordan Nurses and Midwives Council, the Mariam Foundation for support cancer patients, FAJR Scientific representatives from the WHO, UNDP, UNICEF, MSF, ICRC, American University of Beirut, Al Balqa University, Jordan University, University of Geneva and many more. What these organisations have in common is their deep historical, personal and professional connections with Gaza, and their commitment to help rebuild the health system in Gaza over the foreseeable future. Indeed, at the conference, all organisations committed to deliver immediate humanitarian services and substantially contribute to rebuilding efforts in the medium and long term. They also committed in principle to the deployment of staff: for example, two thousand Egyptian medical doctors are on standby to be deployed; fifty British nurses and doctors have gathered enough medicine and equipment to operate in Gaza; and Jordanian engineers aim to enter Gaza to assess and restore the water network and other damaged infrastructure in anticipation of future reconstruction. At the time of writing, the border remains closed, and these commitments remain aspirational. Furthermore, while the meeting showcased the variety of skills and dedication available to help Gazans, the credibility of the coalition, as a humanitarian or health sector actor, will require the essential task of coordinating the actions of such a heterogeneous group of organisations and ensuring they speak with one united voice.

At the Amman conference, a vision for the reconstruction of the Gaza health sector was proposed, with vulnerable groups that need to be prioritised identified [4]. These populations of focus include children (orphans and children with amputations), elderly people, pregnant women, people who have chronic conditions, people who need urgent surgery, healthcare workers, and students in health-related professions. The vision was built mainly on scarce available data and assumptions, according to three different time horizons: first, the immediate humanitarian needs, which are enormous and require actions that go well beyond the health sector; second, a recovery trajectory aligned with available capabilities, in parallel with field hospitals and alternative care delivery models; and third, a comprehensive health system reconstruction in alignment with WHO standards. At all stages, there will be immense challenges, starting with basic needs: for example, safe, clean water sources need to be restored, hospitals and roads need to be cleared from unexploded ordnance, and the safety and security of health professionals need to be ensured. Health needs assessments, both at facility and community levels, will be primordial as data systems have been interrupted, including disease surveillance. This extra support, if security allows, could be delivered inside the Gaza Strip, or through medical evacuations to neighbouring countries, where a large number of coalition-affiliated hospitals have agreed to deliver the required healthcare services. Immediate actions will also focus on public health through vaccinations, nutrition centres, and the supply of water. Two subsequent phases after the emergency phase are planned for the rebuilding: the mid-term recovery phase will focus on restoring the services rather than the infrastructures. This will require field hospitals, mobile clinics, the decontamination of roads and buildings polluted by unexploded ordnance and the rebuilding of a network of primary healthcare facilities as a priority. The long-term reconstruction phase will require the building and equipment of secondary and tertiary hospitals and universities.

Relieving Palestinian healthcare professionals from their duties so that they can rest, recuperate, and receive psychological support will require the deployment of a large number of external healthcare staff. Extra support will also be required for emergency trauma surgery, which is estimated at 11,000 individuals in need of urgent operations – a task that is unfeasible at the moment at such a scale considering the total absence of operational hospitals. Human resources support will also focus on training the next generation of healthcare workers. Coalition members with support from universities in the region committed to supporting the medical education of approximately 300 medical students whose training was interrupted by the current conflict, offering bursaries and support, including mentorship, to finish their internships and residencies in neighbouring countries. In the third phase, the public health system will need to be restored, hospitals will need to be built and staffed and connected with a strong primary healthcare system, and this coalition has the ambition to build a resilient sector that can be sustained over the long term.

While much of the rebuilding of the health sector will focus on technical aspects, it is imperative that the interplay of political, social, economic, and environmental determinants of health be accounted for in any such effort. This includes, as a first step, the opening of the Rafah border to allow humanitarian supply to medicine, equipment and health staff and a cease-fire to ensure the protection of civilians and humanitarian and health personnel. Any response in the short term should also build on existing coordination mechanisms among local stakeholders across sectors and with the traditional humanitarian system (the United Nations agencies and international humanitarian organisations) and should avoid duplication of efforts to optimize limited resources. In the medium term, clarity on the political shape of the governance in Gaza Strip with various political scenarios are envisaged [5], which will help guide the rebuilding of the health system capitalizing on the strengths of the previously existing public health system and emergency response mechanisms. As for the long-term, it will surely require a strong commitment from all, including actors such as this global collective movement with a well-coordinated diaspora, that would help overcome potential “donor fatigue” and local stakeholder exhaustion and trauma.

Already ten years ago, in 2014, extensive destruction of healthcare facilities by the Israeli army required $1 billion to reconstruct Gaza’s fragile health system [6]. It is estimated that it will take at least ten years and $ 15 billion to rebuild the infrastructure damaged during the current war. Planning this reconstruction can already begin, even in midst of ongoing conflict, in order to identify, collect, and coordinate technical resources and financial commitments. But we join the chorus of so many voices in demanding that the cycle of destruction and reconstruction needs to stop, in order that the civilian victims on both sides of this decades-long conflict can live in peace, good health, and dignity.

## Data Availability

NA.
