# Correction: Rebuilding the health sector in Gaza: alternative humanitarian voices

**DOI:** 10.1186/s13031-024-00602-8

**Published:** 2024-06-25

**Authors:** Karl Blanchet, Martine Najem, Lina Shadid, Rouba Ali Fehmi, Fawzi Al Hammouri, Ghassan Saed, khaled J Saleh, Mosab Nasser, Nidal Moukaddam, Jonathan Polonsky, Omar Lattouf

**Affiliations:** 1 Geneva Centre of Humanitarian Studies, Faculty of Medicine, University of Geneva, Geneva, Switzerland; 2 American University of Beirut, Beirut, Lebanon; 3 PWC Lead Healthcare, Dubai, United Arab Emirates; 4 University of Michigan, Ann Arbor, Michigan USA; 5 NAAMA, Amman, Jordan; 6 FAJR Scientific, Ann Arbor, USA; 7 Psychiatry Baylor College of Medicine, Waco, USA; 8 Emory University School of Medicine, NAAMA, Atlanta, USA

**Correction: Conflict and Health (2024)18: 42**

10.1186/s13031-024-00599-0

Following publication of the original article [1], the authors identified an error in the author name of Khaled J Saleh.

The incorrect author name is: Khalid Saleh.

The correct author name is: Khaled J Saleh.

The author group has been updated above and the original article [1] has been corrected.

